# Serum cytokine biomarker panels for discriminating pancreatic cancer from benign pancreatic disease

**DOI:** 10.1186/1476-4598-13-114

**Published:** 2014-05-20

**Authors:** Victoria E Shaw, Brian Lane, Claire Jenkinson, Trevor Cox, William Greenhalf, Christopher M Halloran, Joseph Tang, Robert Sutton, John P Neoptolemos, Eithne Costello

**Affiliations:** 1 NIHR Liverpool Pancreas Biomedical Research Unit, Royal Liverpool and Broadgreen University Hospital NHS Trust, Department of Molecular and Clinical Cancer Medicine, University of Liverpool, Liverpool L69 3GA, UK

**Keywords:** Pancreatic cancer, Biomarker, Cytokine, CA19-9, IP-10, IL-8 and PDGF

## Abstract

**Background:**

We investigated whether combinations of serum cytokines, used with logistic disease predictor models, could facilitate the detection of pancreatic ductal adenocarcinoma (PDAC).

**Methods:**

The serum levels of 27 cytokines were measured in 241 subjects, 127 with PDAC, 49 with chronic pancreatitis, 20 with benign biliary obstruction and 45 healthy controls. Samples were split randomly into independent training and test sets. Cytokine biomarker panels were selected by identifying the top performing cytokines in best fit logistic regression models during multiple rounds of resampling from the training dataset. Disease prediction by logistic models, built using the resulting cytokine panels, was evaluated with training and test sets and further examined using resampled performance evaluation.

**Results:**

For the discrimination of PDAC patients from patients with benign disease, a panel of IP-10, IL-6, PDGF plus CA19-9 offered improved diagnostic performance over CA19-9 alone in the training (AUC 0.838 vs. 0.678) and independent test set (AUC 0.884 vs. 0.798). For the discrimination of PDAC from CP, a panel of IL-8, CA19-9, IL-6 and IP-10 offered improved diagnostic performance over CA19-9 alone with the training (AUC 0.880 vs. 0.758) and test set (AUC 0.912 vs. 0.848). Finally, for the discrimination of PDAC in the presence of jaundice from benign controls with jaundice, a panel of IP-10, IL-8, IL-1b and PDGF demonstrated improvement over CA19-9 in the training (AUC 0.810 vs. 0.614) and test set (AUC 0.857 vs. 0.659).

**Conclusions:**

These findings support the potential role for cytokine panels in the discrimination of PDAC from patients with benign pancreatic diseases and warrant additional study.

## Introduction

Novel biomarkers for use in disease detection and/or treatment are urgently needed to improve outcomes for patients with pancreatic cancer (PDAC) [1,2]. Supplementing current diagnostic modalities with biomarker detection in blood [3] could potentially enhance PDAC diagnosis. At present, the only serum biomarker in routine clinical use for PDAC is CA19-9 [4-6]. The ability of novel biomarkers to accurately detect PDAC depends on their capacity to discriminate PDAC from benign diseases of the pancreas, such as chronic pancreatitis. In addition, a majority of PDAC patients present with tumours involving the pancreatic head, which leads to obstructive jaundice [7]. The differentiation of PDAC in jaundiced patients from benign obstructive jaundice due to choledocholithiasis or chronic pancreatitis is a major clinical challenge.

CA19-9 is a sialyated Lewis blood group cell surface carbohydrate antigen, expressed in normal pancreatic ductal cells in around 95% of the population which express the Lewis antigen glycosyltransferase enzyme. CA19-9 is shed into the general circulation and is commonly used in clinical practice to monitor patients with PDAC [4,5,8-10]. CA19-9 is also secreted in a mucin bound form by the biliary and gallbladder mucosa and is exclusively excreted in bile [11]. Serum levels of CA19-9 are elevated in patients with chronic pancreatitis and benign biliary obstruction to a similar extent as in patients with smaller pancreatic cancers [4,8]. Consequently the overall accuracy of CA19-9 for the diagnosis of PDAC is reduced but there is also the opportunity to enhance the specificity of CA19-9 in combination with other tumour-associated biomarkers [12-17]. Mediators of the tumour microenvironment and the host response [12-15,18] and notably cytokines involved in the immune system, inflammation, tumour development and metastasis [19,20] are emerging as key candidate biomarkers. While single cytokines lack sensitivity and specificity for accurate cancer detection [21], specific combinations may prove valuable as markers.

Cytokine biomarker panels for the discrimination of specific patient groups were selected by identifying the best logistic regression models during multiple rounds of resampling [22] from a training dataset. The resulting optimum panels were evaluated using logistic regression models in both training and independent test sets before further subjecting panels to resampling performance evaluation. We discovered a unique panel of cytokines that improved the performance of CA19-9 for the discrimination of PDAC patients from patients with benign pancreatic disease. Moreover, in the presence of jaundice, whilst CA19-9 offered relatively poor discrimination of PDAC patients from benign disease patients, a panel made up solely of cytokines afforded significantly better discrimination.

## Results

### Cytokine levels in patients diagnosed with PDAC, chronic pancreatitis and benign biliary obstruction and healthy subjects

Filtering of the entire dataset showed that serum levels of nine cytokines, comprising PDGF, IL-1b, IL-1ra, IL-6, IL-8, Eotaxin, IP-10, MCP-1 and MIP-1b, were significantly different between PDAC in comparisons with one or more of the control variants (Table 1). Serum levels of five cytokines, IL-1ra, IL-6, IL-8, IP-10 and MIP-1b, as well as serum CA19-9 levels were significantly increased in PDAC compared to HCs. Of these, CA19-9, IL-8 and IP-10, were also significantly elevated in PDAC compared to patients with CP, whilst a comparison of PDAC versus BBO revealed significant increases in serum levels of Eotaxin, IL-1b, MIP-1b and PDGF (Table 1). Serum levels of CA19-9, IL-8, IP-10, MIP-1b and PDGF, were significantly elevated in patients with PDAC compared to patients with benign disease (Table 1). Comparison of serum cytokine levels in subjects with obstructive jaundice showed that IL-8, IP-10, MIP-1b, PDGF and CA19-9 were all significantly elevated in PDAC compared to controls (Table 2). The circulating median levels of cytokines and CA19-9 (i.e. un-normalised) are shown in Additional file 1: Table S1. Spearman’s Rank analysis of the cytokines incorporated into panels and CA19-9 for each group showed a maximum Rho of 0.361, indicating no correlation between age and analyte level.

**Table 1:** Normalised cytokine data in the combined training and test datasets

	**Median (95% CI)**					**P value**			
**Analyte**	**PDAC (n = 127)**	**HC (n = 45)**	**CP (n = 49)**	**BBO (n = 20)**	**CP + BBO (n = 69)**	**PDAC vs. HC**	**PDAC vs. CP**	**PDAC vs. BBO**	**PDAC vs. CP + BBO**
**CA19-9**	0.01	-5.01	-2.7	-0.82	-2.25	<0.001	<0.001	NS	<0.001
	(-1.0 to 0.2)	(-5.4 to -4.6)	(-3.5 to -2.2)	(-2.6 to 0.5)	(-3.2 to -1.6)				
**Eotaxin**	0.48	0.23	0.53	-0.08	0.45	NS	NS	<0.05	NS
	(0.3 to 0.6)	(-0.2 to 0.5)	(0.2 to 0.8)	(-0.5 to 0.7)	(-0.03 to 0.7)				
**IL-1b**	0.23	0.16	0.27	0.14	0.17	NS	NS	<0.05	NS
	(0.1 to 0.4)	(0.08 to 0.3)	(0.04 to 0.6)	(-0.5 to 0.5)	(0.05 to 0.4)				
**IL-1ra**	0.72	0.18	0.78	0.06	0.59	<0.05	NS	NS	NS
	(0.4 to1.0)	(-0.3 to 0.4)	(0.4 to 1.3)	(-1.5 to 1.5)	(0.2 to 1.0)				
**IL-6**	1.25	0.41	1.65	1.78	1.71	<0.001	NS	NS	NS
	(1.0 to 1.5)	(0.1 to 0.7)	(1.2 to 2.1)	(1.0 to 2.5)	(1.2 to 2.0)				
**IL-8**	0.95	0.02	0.54	0.38	0.51	<0.001	<0.001	NS	<0.001
	(0.8 to1.2)	(-0.3 to 0.06)	(0.3 to 0.8)	(-0.4 to 1.1)	(0.3 to 0.8)				
**IP-10**	-1.85	-2.99	-2.6	-2.52	-2.59	<0.001	<0.001	NS	<0.001
	(-2.0 to -1.7)	(-3.3 to -2.7)	(-2.9 to -2.3)	(-2.9 to -1.5)	(-2.8 to -2.3)				
**MCP-1**	0.31	0.44	0.92	0.64	0.81	NS	NS	NS	NS
	(0.2 to 0.6)	(-0.02 to 0.6)	(0.3 to 1.2)	(-0.2 to 1.6)	(0.3 to 1.2)				
**MIP-1b**	0.31	-0.45	0.02	-0.28	-0.06	<0.001	NS	<0.05	<0.01
	(0.2 to 0.4)	(-0.6 to -0.2)	(-0.3 to 0.4)	(-0.5 to 0.2)	(-0.3 to 0.2)				
**PDGF**	0.44	0.31	0.21	-0.62	0.09	NS	NS	<0.01	<0.01
	(0.2 to 0.5)	(0.1 to 0.4)	(-0.02 to 0.5)	(-1.5 to 0.2)	(-0.18 to 0.4)				

Median normalised log_2_ transformed data are presented for each analyte; the 95% CI are given in brackets. PDAC = Cancer, CP = Chronic Pancreatitis, BBO = Benign Biliary Obstruction. Wilcoxon signed rank tests were used to generate p-values for group comparisons. NS - non-significant.

**Table 2:** Cytokine data from patients with biliary obstruction (cancer and benign) in the combined training and test datasets

**Analyte**	**Median (95% CI) PDAC with biliary obstruction (n = 83)**	**Median (95% CI) BBO plus CP with biliary obstruction (n = 27)**	**P Value PDAC with biliary obstruction vs. Obstructed BBO plus CP with biliary obstruction**	**Median (95% CI) PDAC without biliary obstruction (n = 44)**	**P value PDAC with biliary obstruction vs. PDAC without biliary obstruction PDAC**
**CA19-9**	0.16	-1.10	<0.05	-1.06	<0.05
	(-0.6 to 0.4)	(-2.3 to -0.5)		(-1.8 to 0.1)	
**Eotaxin**	0.48	0.40	NS	0.51	NS
	(0.2 to 0.6)	(-0.3 to 0.7)		(0.2 to 0.8)	
**IL-1b**	0.23	0.25	NS	0.24	NS
	(0.1 to 0.4)	(0.03 to 0.4)		(0.002 to 0.4)	
**IL-1ra**	0.72	0.33	NS	0.79	NS
	(0.4 to 1.0)	(-0.8 to 1.0)		(0.2 to 1.3)	
**IL-6**	1.38	1.97	NS	0.92	NS
	(1.2 to 1.7)	(1.0 to 2.4)		(0.7 to 1.3)	
**IL-8**	0.96	0.39	<0.05	0.89	NS
	(0.8 to 1.4)	(0.2 to 1.1)		(0.5 to 1.2)	
**IP-10**	-1.79	-2.59	<0.05	-2.06	NS
	(-2.0 to -1.6)	(-2.8 to -2.2)		(-2.5 to -1.7)	
**MCP-1**	0.26	0.56	NS	0.40	NS
	(0.05 to 0.6)	(0.001 to 1.4)		(0.08 to 0.8)	
**MIP-1b**	0.34	-0.06	<0.05	0.29	NS
	(0.2 to 0.5)	(-0.5 to 0.3)		(-0.09 to 0.35)	
**PDGF**	0.42	-0.27	<0.01	0.51	NS
	(0.2 to 0.5)	(-0.9 to 0.4)		(0.1 to 0.7)	

Median normalised log_2_ transformed data are presented for each analyte; the 95% CI are given in brackets. PDAC = Cancer, CP = Chronic Pancreatitis, BBO = Benign Biliary Obstruction. Wilcoxon signed rank tests were used to generate p-values for group comparisons. NS - non-significant.

### Classification model to distinguish patients with PDAC from healthy subjects

**Figure 1:**
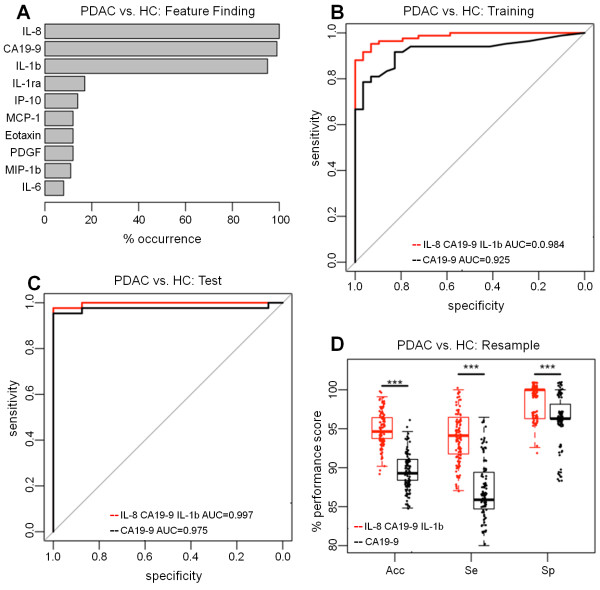
**Classification of patients with PDAC vs. healthy control individuals. A.** Feature finding showing the percentage occurrence of cytokines in models following resampling of training data for PDAC vs. Healthy Control. **B.** Training set ROC Curves for PDAC vs. Healthy Control for a panel of IL-8, CA19-9 and IL-1b versus CA19-9 alone. **C.** Test set ROC curves for PDAC vs. Healthy Control for the panel of IL-8, CA19-9 and IL-1b versus CA19-9. **D.** The resampling performance of the panel of IL-8, CA19-9 and IL-1b versus CA19-9 for the classification of PDAC vs. Healthy Control. The accuracies, sensitivities and specificities of the panel compared to CA19-9 alone over 100 patient-balanced resamples is shown. The quantiles of the performance indicators are summarised as boxplots with individual points superimposed and the significance of Friedman test comparisons is indicated (***p <0.001).

In the training dataset (84 PDAC, 29 HCs), serum CA19-9 had a very high performance in distinguishing patients with PDAC from healthy subjects (AUC = 0.925, CI = 0.876-0.974). Serum levels of IL-8 and IL-1b were also both found with high frequency in top ranked models of the training set in successive resamples (100% and 95% respectively) as well as CA19-9 (99%) (Figure 1A). The combined panel comprising IL-8, IL-1b and CA19-9 showed a statistically significant improvement in accuracy over CA19-9 alone (AUC = 0.984, CI = 0.968-1.00 vs. AUC = 0.925, CI = 0.876-0.974; p = 0.004; Figure 1B), although diagnostic improvement of the panel over CA19-9 was not statistically significant in the test set (PDAC = 43, HC = 16) (AUC = 0.997, CI = 0.990-1.00 vs. AUC = 0.975, CI = 0.932-1.00) (Figure 1C). Resampling the combined dataset (PDAC = 127, HC = 45) 100 times however, showed a statistically significant improved accuracy for the panel compared to CA19-9 alone (median = 94.6%, IQR = 93.8-96.4% vs. median = 89.3%, IQR = 88.4-91.1% respectively; Friedman test p < 0.001). The SN (median = 94.1%, IQR = 91.8-96.4%, versus 85.9%, IQR = 84.7-89.4% respectively) and SP (median = 100%, IQR = 96.3-100% versus median = 96.3%, IQR = 96.3-97.2%) of the panel on resampling were also significantly higher than resampled CA19-9 alone (both Friedman test p < 0.001; Figure 1D).

### Classification model to distinguish patients with PDAC from patients with benign disease

**Figure 2:**
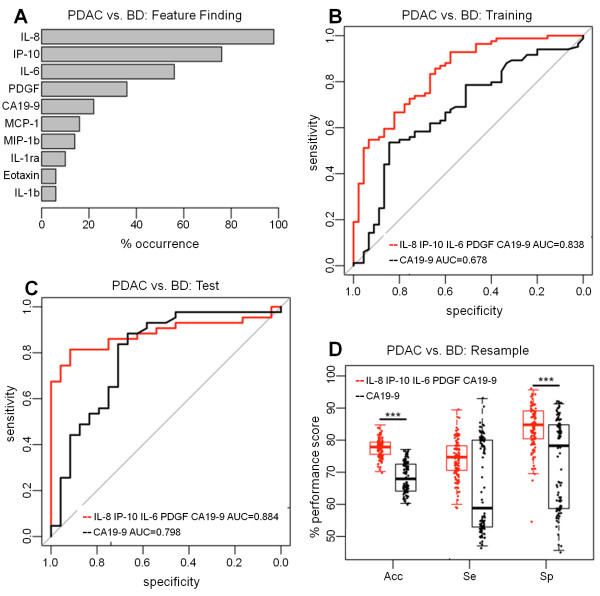
**Classification of patients with PDAC vs. patients with benign disease. (A)** Feature finding results showing the percentage occurrence of cytokines in models following resampling of training data for PDAC vs. benign disease. **(B)** Training set ROC Curves for PDAC vs. benign disease for a panel of IL-8, IP-10, IL-6, PDGF and CA19-9 versus CA19-9 alone. **(C)** Test set ROC curves for PDAC vs. benign disease for the panel of IL-8, IP-10, IL-6, PDGF versus CA19-9. **(D)** The resampling performance of the panel of IL-8, IP-10, IL-6, PDGF and CA19-9 versus CA19-9 for the classification of PDAC vs. benign disease. The accuracies, sensitivities and specificities of the panel compared to CA19-9 alone over 100 patient-balanced resamples is shown. The quantiles of the performance indicators are summarised as boxplots with individual points superimposed and the significance of Friedman test comparisons is indicated (***p < < 0.001).

Model building using the training set (PDAC = 84, benign disease = 45) to distinguish PDAC patients from patients with benign pancreatic disease showed that the most frequent cytokines in top ranked resampled models were IL-8 (98%), IP-10 (76%), IL-6 (56%) and PDGF (36%) plus CA19-9 (22%) (Figure 2A). As a panel, these cytokines demonstrated improved diagnostic performance over CA19-9 alone (AUC = 0.838; CI = 0.768-0.909 vs. AUC = 0.678; CI = .579-0.776; p < 0.001) (Figure 2B) with significantly improved SN (median = 92.9%, CI = 85-97% vs. median = 53.6%, CI = 42-65%, respectively, p < 0.001), but significantly reduced SP (median = 57.8%, CI = 42-72% vs. median = 84.4%, CI = 71-94% respectively, p = 0.008). Using the independent test data (PDAC = 43, benign disease = 24), the panel showed improved performance over CA19-9 alone (AUC = 0.884, CI = 0.802-0.966 vs. AUC = 0.798, CI 0.676-0.919 respectively; p = 0.058) (Figure 2C) with a median SN of 81.4% (CI = 67-92%) and SP of 91.7% (CI = 73-99%) for the panel compared to 88.4% (CI = 75-96%) and 66.7% (CI = 45-84%) respectively for CA19-9 alone. The inconsistency in the outcomes with training and test set data here illustrate the problems associated with diagnostic performance assessment using single data splits.

Resampling of the combined dataset (PDAC = 127, benign disease n = 69) (Figure 2D) generated 100 independent optimum values for SN, SP and accuracy and showed that the accuracy of the cytokine panel (median = 77.8%, IQR = 75.6-79.4%) was significantly improved compared to CA19-9 alone (median = 67.9%, IQR = 64.1-72.5%; Friedman test p < 0.001). The SP of the cytokine panel (median = 84.8%, IQR = 80.4-89.1%) was significantly better than that of CA19-9 alone (median = 78.2%, IQR = 58.7-84.8%; Friedman test p < 0.001), while the improvement in SN was not statistically significant (median = 74.7%, IQR 70.6-77.9% vs. median = 58.8%, IQR = 52.9-80.0% respectively; Friedman test p = 0.071) (Figure 2D).

### Classification model to distinguish patients with PDAC from patients with chronic pancreatitis

**Figure 3:**
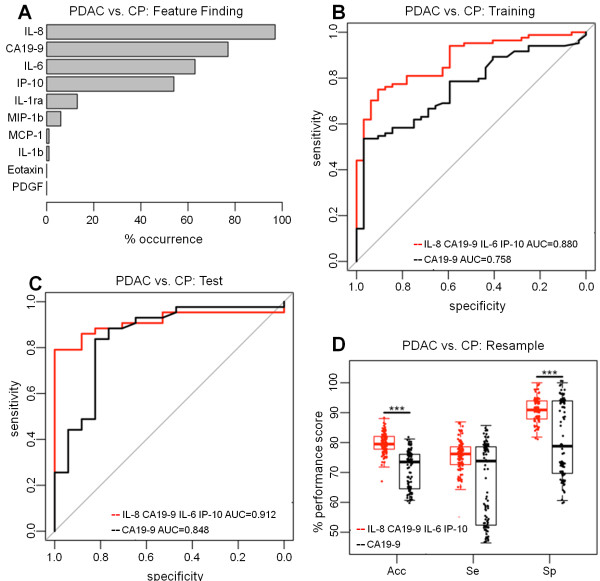
**Classification of patients with PDAC vs patients with chronic pancreatitis. (A)** Feature finding results showing the percentage occurrence of cytokines in models following resampling of training data for PDAC vs. chronic pancreatitis. **(B)** Training set ROC Curves for PDAC vs. chronic pancreatitis for a panel of IL-8, CA19-9, IL-6, IP-10 versus CA19-9 alone. **(C)** Test set ROC curves for PDAC vs. chronic pancreatitis for the panel of IL-8, CA19-9, IL-6 and IP-10 and CA19-9 alone. **(D)** The resampling performance of the panel of IL-8, CA19-9, IL-6, IP-10 versus CA19-9 for the classification of PDAC vs. chronic pancreatitis. The accuracies, sensitivities and specificities of the panel compared to CA19-9 alone over 100 patient-balanced resamples is shown. The quantiles of the performance indicators are summarised as boxplots with individual points superimposed and the significance of Friedman test comparisons is indicated (***p < < 0.001).

Optimum cytokine combinations for the discrimination of PDAC from CP with the training set (PDAC = 84 and CP = 32) showed that the most frequent cytokines in resampled models were IL-8 (97%), IL-6 (63%) and IP-10 (54%) as well as CA19-9 (77%) (Figure 3A). A panel comprising IL-8, IL-6 and IP-10 with CA19-9, showed a significantly improved diagnostic performance with the training data over CA19-9 alone (AUC = 0.880, CI = 0.818-0.943 vs. AUC = 0.758, CI = 0.668-0.849; p = 0.005) (Figure 3B). The SN of the panel (median = 75.0%, CI = 64-84%) was significantly better than CA19-9 alone (median = 53.6%, CI = 42-65%; p < 0.001) whilst maintaining a high SP (median = 90.6%, CI = 75-98% vs. median = 96.9%, CI = 84-100%, respectively). In the independent test set (PDAC = 43, CP = 17), the AUC achieved with the panel was greater than that of CA19-9 (0.912; CI 0.838-0.987 vs. AUC 0.848; CI 0.728-0.968, respectively) (Figure 3C).

Resampling the combined dataset (PDAC = 127 and CP = 49) revealed a significant improvement in accuracy with the cytokine panel (median = 79.5%, IQR = 77.8-82.1%) compared to CA19-9 (median = 73.5%, IQR = 64.7-76.1%; Friedman test p < 0.001) (Figure 3D). The SP was also significantly better (median = 90.9%, IQR = 87.9-93.9% vs. median = 78.8%, IQR = 69.7-93.9% respectively; Friedman test p < 0.001) with no significant difference in SN estimates (median = 76.2%, IQR = 72.6-78.6% vs. median = 73.8%, IQR = 52.3-78.6%, respectively; Friedman test p = 0.053).

### Classification model in patients with obstructive jaundice to distinguish patients with PDAC from patients with benign disease

**Figure 4:**
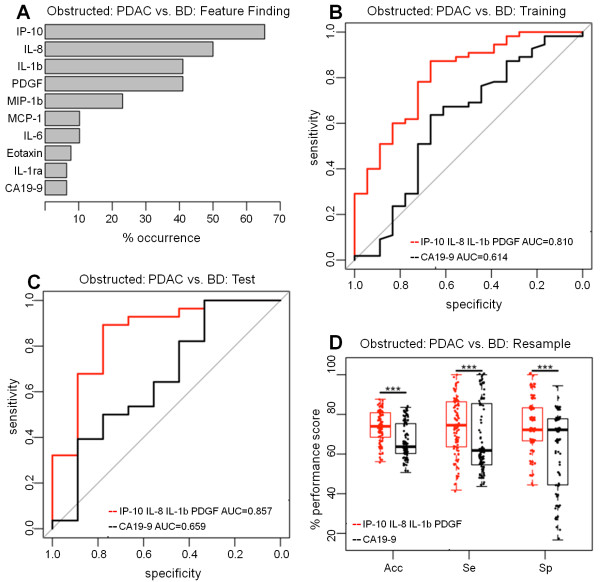
**Classification of patients with PDAC in the presence of jaundice vs. patients with benign disease in the presence of jaundice. (A)** Feature finding results showing the percentage occurrence of cytokines in models following resampling of training dataset for PDAC patients with high bilirubin vs. patients with benign disease and high bilirubin. **(B)** Training set ROC Curves for PDAC patients with high bilirubin vs. patients with benign disease and high bilirubin for a panel of IP-10, IL-8, IL-1b, PDGF and CA19-9 versus CA19-9 alone. **(C)** Test set ROC curves for PDAC patients with high bilirubin vs. patients with benign disease and high bilirubin for the panel of IP-10, IL-8, IL-1b, PDGF, CA19-9 compared to CA19-9 alone. **(D)** The resampling performance of the panel and CA19-9 for the classification of high bilirubin PDAC vs. high bilirubin benign disease. The accuracies, sensitivities and specificities of the panel IP-10, IL-8, IL-1b, PDGF, CA19-9 compared to CA19-9 alone over 100 patient balanced resamples is shown. The quantiles of the performance indicators are summarised as boxplots with individual points superimposed and the significance of Friedman test comparisons is indicated (***p < < 0.001).

The training dataset of patients with serum bilirubin levels ≥ 20 μmol/L (PDAC = 55, benign disease = 18) was resampled and the top ranked regression models most frequently featured were IP-10 (65.4%), IL-8 (50.0%), IL-1b (41.0%) and PDGF (41.0%) whilst CA19-9 was the feature of lowest importance (6.1%) (Figure 4A). The cytokine panel (IP-10,IL-8,IL-1b and PDGF) was significantly more accurate than CA19-9 (AUC = 0.810, CI = 0.693-0.927 vs. AUC = 0.614, CI = 0.445-0.782; p = 0.023) (Figure 4B) with significantly improved SN (median = 87.3%, CI = 76-95% vs. median = 63.6%, CI = 50-76% respectively; p < 0.001) and unchanged SP (median = 66.7%, CI = 41-87% vs. median = 66.7%, CI = 41-87%, respectively). The test set of samples with serum bilirubin levels ≥ 20 μmol/L (PDAC = 28, benign disease = 9) also showed diagnostic improvement (AUC = 0.857, CI = 0.699-1 vs. AUC = 0.659, CI = 0.426-0.892 respectively) (Figure 4C).

Resampling the combined dataset of patients with serum bilirubin levels ≥ 20 μmol/L (PDAC = 83 and benign disease = 27) generated 100 independent optimum values and showed significantly greater accuracy with the panel over CA19-9 (median = 73.9%, IQR = 68.5-80.8% vs. median = 63.7%, IQR = 60.3-75.3%, respectively; Friedman test p < 0.001) (Figure 4D). In particular, the SN for the cytokine panel was significantly higher than that of CA19-9 alone (median = 74.5%, IQR = 63.6-85.9% vs. median = 61.8, IQR = 54.5-85.4%, respectively; Friedman test p = 0.002).

### Ability to detect resectable PDAC and advanced PDAC cases

The study included samples from both resectable and advanced PDAC cases (Additional file 1: Table S1). Binomial logistic modelling did not identify cytokines that distinguished between these two disease categories in either the training or test sets. However, post hoc tests showed that advanced and resectable PDAC were equally likely to be detected in our models as there was no significant difference in the proportion of advanced and resectable cases detected in pooled training and test set data (Pearson chi squared test for equality of proportion of detections of advanced and resectable PDAC at df =1; PDAC vs. HC, p = 0.997; PDAC vs. Benign Disease, p = 0.417; PDAC vs. CP, p = 0.704 and PDAC with obstructive jaundice vs. Benign Disease with obstructive jaundice, p = 0.892).

## Discussion

The accurate diagnosis of PDAC against a complex range of primary secondary and even tertiary health care scenarios remains a major clinical challenge. More specifically the clinical settings in which biomarker panels may be used to facilitate accurate diagnosis are variable, depending on whether the disease is asymptomatic or symptomatic, and whether jaundice is present or absent. Thus, improving diagnosis involves discriminating patients with pancreatic cancer from patients with benign diseases of the pancreas or the biliary system. In this study, we used advance logistic modeling to determine how reliably distinct combinations of cytokines could facilitate differential pancreatic cancer diagnosis.

The performance of CA19-9 for the discrimination of PDAC patients from healthy subjects is variable, with some studies reporting just acceptable AUC values of 0.83-0.84 [15,23], while one study an accuracy as high as AUC = 0.90 [24]. In this study we observed a very strong performance from CA19-9 compared to healthy controls, providing an AUC >0.92 in both training and test sets and very high median resampled estimates of optimum SN and SP were 85.9% and 96.3% respectively. The addition of two cytokines (IL-8 and IL-1b) to CA19-9 in a panel enhanced the performance of CA19-9, increasing the median resampled accuracy from 89% to 95% while the median resampled SN and SP was increased to 94% and 100%, respectively. This supports the finding by Ebrahimi *et al.*[25] who found that IL-8 was increased in serum from PDAC patients compared to healthy controls, as was IL-1ra and IL-6, although these cytokines did not form part of the final panel.

The cytokines IL-8, IP-10, IL-6 and PDGF emerged as the strongest candidates in discriminating patients with PDAC from patients with benign pancreatic disease and combining these cytokines with CA19-9 afforded enhanced discrimination. The inconsistent outcomes with the training and test set data in this case illustrate the potential problems of overfitting and bias associated with single data splits, and supports the use of resampling for a more robust estimate of performance. It is notable that the SN and SP estimates with CA19-9 during resampling were bi-modal while the panel-derived estimates were uniformly distributed (Figure 2D). This suggested that the performance of CA19-9 alone was less uniform and therefore less reliable than the performance of the cytokine panel. Interestingly, a similar bi-modal distribution, resulting in an increase in the range of SN and SP estimates with CA19-9 compared to cytokine panels, was observed with resampled PDAC versus CP (Figure 3D), and with resampled High Bilirubin PDAC versus High Bilirubin Benign Disease patients (Figure 4D). This suggests that the panels in general provided a more reliable performance than CA19-9 alone.

Whilst CA19-9 levels were significantly raised in patients with biliary obstruction as might be expected [10,26,27] the levels of individual cytokines in cancer patients were not associated with jaundice. In discriminating jaundiced patients with PDAC from jaundiced patients with benign pancreatic disease, CA19-9 alone, performed poorly. IP-10, IL-8, IL-1b and PDGF, which were all significantly elevated in jaundiced patients with PDAC compared to jaundiced patients with benign disease, enabled significantly better discrimination of these two groups when used in combination. Even though obstructive jaundice occurs relatively late in pancreatic cancer, this can wane so more efficient and accurate differential diagnosis of these patients is of considerable importance in routine clinical practice. IL-8 levels are elevated in PDAC patients [15,28] and this pro-inflammatory cytokine was a prominent feature as a discriminator for PDAC in all of the panels in this study. It is produced in monocytes and endothelial cells, and a variety of tumours [28-30] and high serum levels of IL-8 in PDAC are linked to poor survival [31].

IP-10 featured in all disease comparison panels for diagnosis, consisitent with a previous small study in PDAC [32] and a study in colorectal cancer [33]. Increased expression of IP-10 and its receptor CXCR3 have also been associated with several advanced human cancers, including ovarian cancer, malignant melanoma, mutliple myeloma and basal cell carcinoma [34]. Whilst IP-10 generally performed well in the discrimination of PDAC from patients with benign disease, it was the best performing analyte for the discrimination of PDAC patients with jaundice from patients with benign disease in the presence of jaundice. This pro-inflammatory chemokine has also been shown to be secreted from several cell types in response to IFN-γ, and attracts activated lymphocytes, monocytes and NK cells to sites of inflammation [34], inhibits angiogenesis and promotes the survival and proliferation of tumour-specific T-cells [34-36].

IL-6 levels were observed to be significantly elevated in patients with PDAC compared to healthy controls, although it did not perform well enough to be part of the cytokine panel [25,37,38]. This cytokine was important in the discrimination of PDAC from chronic pancreatitis where it was the third most important analyte after IL-8 and CA19-9, during feature finding. It had limited value in distinguishing PDAC patients from patients with jaundice due to benign disease. IL-6 is a pro-imflamatory cytokine that it involoved in the recruitment of neutrophils and stimulating T-cell proliferation and migration [39]. High serum levels of IL-6 have been shown in many different cancer types, and positive associations with tumour stage, size and disease progression have been reported [39]. PDGF levels were not significantly different between patients with PDAC, patients with chronic pancreatitis and healthy subjects. Significantly decreased serum levels of PDGF were observed in patients with jaundice due to benign disease and may account for the discrimination of cancer patients from patients with biliary obstruction.

Other groups have examined the ability of cytokine panels to detect PDAC. Zeh *et al.*[40] used LabMAP serum technology with classification trees to identify panels to distinguish pancreatic cancer patients from chronic pancreatitis patients or control subjects. Interestingly their study identified IP-10 and IL-8 as having ability to discriminate PDAC from these two groups [40]. This is consistent with our study in which IL-8 was the best performing cytokine to distinguish PDAC cases from healthy controls and IP-10 and IL-8 were both featured in models distinguishing pancreatic cancer from benign disease. More recently, Dima *et al.*[31] explored the levels of circulating inflammatory cytokines in a small number of pancreatic cancer patients and controls. The study identified high levels of IL-6 in PDAC cases compared to chronic pancreatitis patients and elevated IL-10 and TNFα in PDAC cases compared to healthy subjects [31].

This study has a number of limitations. The sample sizes for investigating the effect of obstructive jaundice on performance were small, especially in the test datasets. In addition, the study suffered the loss of a number of analytes due to large coefficients of variance. Analytes such as IL-2, IL-15, and MIP-1a were present at very low concentrations and undetectable in more than half of the subjects studied. As expected the coefficient of variation was higher for analytes measured at lower concentrations on the Luminex platform compared to those measured at higher concentrations [41]. The diagnostic potential of these analytes should be tested under conditions sensitive to lower concentrations. Finally, we have confined this study to evaluating the potential of serum cytokine panels for pancreatic cancer diagnosis. The relationship between serum cytokine levels and prognosis is worthy of study, although lies outside the scope of this manuscript.

## Conclusions

In summary, we show that for the discrimination of patients with PDAC from patients with benign disease, combining IL-8, IP-10, IL-6 and PDGF with CA19-9 was better than using CA19-9 alone. Moreover, whilst CA19-9 was ineffective at discriminating between jaundiced PDAC patients versus jaundiced controls, a panel containing IP-10, IL-8, IL-1b and PDGF provided good discrimination. These findings support the potential role for specific cytokines in the differential diagnosis of pancreatic cancer and warrants additional study.

## Methods

Blood samples using standard operating procedures were obtained from pre-surgical resection (resectable) or by-pass (advanced) patients with histologically confirmed PDAC, histologically confirmed chronic pancreatitis (CP), benign biliary obstruction (BBO) or from healthy controls (HC). Resectable PDAC patients had normal tissue plane between tumour and vessels and no evidence of metastatic disease or tumour abutment less than 180° of the SMA or coeliac axis, venous involvement up to 2 cm occlusion of the SMV, PV or SMV-PV confluence with no evidence of metastatic disease [42-44]. All patients gave written informed consent using approved ethics protocols, at the Royal Liverpool University Hospital.

### Serum collection

Blood was collected in Sarstedt Monovette Serum Z tubes (Sarstedt Ltd, Leicester, UK) and allowed to coagulate for 30 minutes before centrifugation at 800 × g for 10 min. The serum fraction was aliquotted into cryotubes and stored at -80°C. CA19-9 levels were measured using ELISA (Human Pancreatic & GI Cancer ELISA Kit, Alpha Diagnostics International, San Antonio, Tx, USA). Pre-operative total serum bilirubin (μmol/L) (Roche Modular SWA) was measured in the hospital Clinical Biochemistry Department.

### Measurement of cytokines

The serum levels of 27 cytokines, chemokines and growth factors from patients and healthy subjects were measured blindly in duplicate using a commercially available Bio-Plex Pro 27 Plex Human Cytokine, Chemokine and Growth Factor Assay (Bio-Rad Laboratories Ltd, Hercules, CA, USA), on the Bio-Plex 200 System, with initial data analysis to measure concentration performed using Bio-Plex Manager 5.0 Software. Briefly, serially diluted standards (50 μL) and test serum, diluted 1 in 4 in sample diluent, (50 μl) was added to a microfilter plate containing antibody-coupled beads for each of the 27 analytes. The microfilter plate was incubated at room temperature on a plate shaker at 900 rpm for 1 minute followed by 300 rpm for 30 minutes. Following washing by vacuum filtration the secondary antibodies (25 μL) were added and the microfilter plate incubated as before. The microfilter plate was washed again and Streptavidin-PE (50 μL) was added and the plate incubated at room temperature on a plate shaker at 900 rpm for 1 minute followed by 300 rpm for 15 minutes. Assay buffer (125 μL) was added to each well of the microfilter plate before being read on the Bio-Plex 200 machine. Fluorescent intensities obtained for the test samples were read from the standard curve to give pg/mL values for each of the 27 cytokines, chemokines and growth factors. Ten assay plates were used to generate training data from 158 individuals, and test data from 83 individuals. To assess inter-plate variation, 6 individual samples were measured across triplicate plates; and at least one aliquot of the same PDAC patient was assayed on every plate for internal control purposes.

### Patient groups

The training set consisted of samples from 158 subjects, 84 patients with PDAC, 45 patients with benign pancreatic disease (32 with CP and 13 with BBO due to gall stones) and 29 HCs. The serum bilirubin level of patients was recorded in all cases as either low (<20 μmol/L; upper level of normal for our Centre) or high (>20 μmol/L). In the training set there were 73 (46.2%) patients with high bilirubin, 55 with PDAC and 18 with BBO including 5 with CP. The independent test set consisted of samples from 83 subjects, 43 patients with PDAC, 17 with CP, 7 with BBO, and 16 HCs. In the test set there were 37 (44.6%) patients with high bilirubin, 28 with PDAC and 9 patients with BBO (including 2 with CP). The clinical characteristics of the training and test study populations are provided in Table 3, with further specific characteristics of cancer patients, separated into resectable and advanced categories, provided in Additional file 2: Table S2. The median age of healthy control subjects in the training set was 44 years compared to a median age of 66 years for the PDAC patients and the median age of healthy control subjects in the test set was higher at 56.5 years.

**Table 3:** Clinical characteristics of respective training and test study populations

**Training set n = 158**	**Age (years) Median (95% CI)**	**Gender Male: Female**	**CA19-9 (KU/L) Median (95% CI)**	**Bilirubin (μmol/L) Median (95% CI)**
**PDAC (n = 84)**	66 (64.0-69.0)	47:37	137.5 (62.0-161.4)	31 (24.5-54.3)
**CP (n = 32)**	53 (48.0-58.6)	20:12	24.5 (11.0-41.0)	7 (6.0-10.0)
**BBO (n = 13)**	72 (60.7-77.4)	11:2	89 (13.5-228.3)	69 (11.0-108.6)
**HC (n = 29)**	44 (29.9-54.0)	14:15	4 (2.0-6.0)	NA
**Test Set n = 83**	**Age (years) Median (95%CI)**	**Gender Male: Female**	**CA19-9 (KU/L) Median (95%CI)**	**Bilirubin (μmol/L) Median (95% CI)**
**PDAC (n = 43)**	68 (64.2-71.8)	24:19	116 (54.9-164.4)	51 (19.6-114.0)
**CP (n = 17)**	53 (45.1-62.0)	10:7	12 (8.0-24.9)	6 (5.0-10.0)
**BBO (n = 7)**	64 (38.4-78.5)	3:4	37 (4.5-181.3)	100 (44.9-374.9)
**HC (n = 16)**	56.5 (36.7-62.1)	9:7	3 (2.6-8.4)	NA

PDAC = Cancer, CP = Chronic Pancreatitis, BBO = Benign Biliary Obstruction, HC = Healthy Controls.

### Data filtering, normalisation

Cytokines with internal control measurements with a coefficient of variance > 50% were removed from the dataset. Cytokine concentrations (pg/mL) less than the lower limit of detection were set to 0.001. The remaining cytokines, along with CA19-9, were used in predictor model building. Normalisation between plates was undertaken by dividing raw cytokine data by the plate-specific internal control value for each cytokine. Normalised data were log_2_ transformed for statistical analysis. The Shapiro-Wilk test with cut-off of p < 0.05 was applied to test the null hypothesis that data were normally distributed. Two-sample Wilcoxon signed rank (Mann–Whitney) tests with a cut-off of p < 0.05 were applied to test the null-hypothesis that the medians of groups with non-parametric (non-normal) distribution were the same. Following normalisation and log_2_ transformation, the class labels (e.g. PDAC, CP, BBO or HC) were added to the data set.

### Building statistical predictor models

Statistical modelling was performed in 64-bit R (2.15.1; R Development Core Team 2012). The training dataset was used to construct binomial logistic models, using the R function glm, to discriminate the following patient groups: PDAC versus HC; PDAC versus Benign Disease (combined CP and BBO patients) PDAC versus CP and PDAC in the presence of jaundice versus Benign Disease in the presence of jaundice.

Cytokines for inclusion in models were selected using repeated (n = 100) balanced splitting of the training set, tabulating the optimal cytokine panel for each model and then ranking cytokines. Feature finding at each data split was performed by complete enumeration of all possible logistic regression models by Morgan-Tatar search, selecting the highest ranked model as scored by Bayesian information criterion using the bestglm package in R. Cytokines were selected if they achieved a regression p < 0.05 in the top ranked model. Logistic predictor models fitted to data in this way consisted of a probability function (i.e. a predictor of class membership) of the form *exp(z)/1 + exp(z)* where *z* is a specific linear combination of coefficients, such that *z = β*_*0*_ *+ β*_*1*_*x*_*1*_ *+ β*_*2*_*x*_*2*_…, where *β*_*i*_ are cytokine specific coefficients found by regression (*β*_*o*_ being the intercept) and *x*_*i*_ are sample cytokine specific values. Classification performance was assessed by Receiver Operating Characteristic (ROC) analysis using the pROC [45] and ROCR [46] packages from R to find the optimum sensitivity (SN), specificity (SP) and Area Under Curve (AUC). Comparisons of AUCs of cytokine panel models versus CA19-9 alone were performed using a one-tailed De Long’s test. The classification performance of models developed with the training data was assessed by internal validation and independently using the test set data. Confidence intervals (95%) for the SN and SP of individual diagnostic tests were calculated as exact binomial proportion intervals using the R package epiR. The McNemar test was used to compare SN and SP of paired diagnostic tests using the R package DTComPair [47].

### Resampled estimates of model performance

The robustness of the classification models developed with training data was evaluated by repeatedly resampling data from the combined training and test cohorts. The combined dataset was split randomly 100 times at a ratio of 2 to 1 into resampled training and test sets, taking care to maintain the proportionate class composition of the resampled training and test sets (i.e. the ratio of cancer: control). At each split, the resampled training dataset was used to build a classification model using the cytokine panel identified during binomial logistic modelling of the original training sets. The ability of each model to classify the resampled test data was recorded. ROC analysis was performed and the SN and SP values, at the optimum accuracy, of each resampled test set were recorded. Thus, each resampled model generated 100 independent estimates of SN, SP at optimised accuracy, which were used to calculate median SN/SP and median accuracy values. Since the distribution of prediction error of resampled models was non-normal and prediction errors with the same test set but different models are not independent, the null hypothesis of equality of prediction error distribution of different models was tested using the Friedman test.

## Competing interests

The authors declare that they have no competing interests.

## Authors’ contributions

VES and CJ carried out cytokine luminex analysis of patient serum samples. VES, EC and BL performed statistical and bioinformatics analysis of cytokine data. TC advised on statistical analysis. VES and EC conceived of the experiments. All authors participated in study design, coordination and helped to draft the manuscript. All authors read and approved the final manuscript.

## Supplementary Material

Additional file 1: Table S1 Circulating cytokine levels in the combined training and test datasets. Click here for file

Additional file 2: Table S2 Patient characteristics of Resectable and Advanced PDAC patients. Click here for file
