# Supplementary material for: Serum cytokine biomarker panels for discriminating pancreatic cancer from benign pancreatic disease
Source: Mol Cancer. 2014 May 20;13:114. doi: 10.1186/1476-4598-13-114 (PMC4032456; doi:10.1186/1476-4598-13-114)
Supplement: Additional file 1: Table S1 — Circulating cytokine levels in the combined training and test datasets. [file 1476-4598-13-114-S1.docx]

**Additional file 1: Table S1.** Circulating cytokine levels in the combined training and test datasets.

|  | Median (95% CI) | | | | | | | |
| --- | --- | --- | --- | --- | --- | --- | --- | --- |
| Analyte | **Median (95% CI)**  **PDAC**  **(n=127)** | **Median (95% CI)**  **HC**  **(n=45)** | **Median (95% CI)**  **CP**  **(n=49)** | **Median (95% CI)**  **BBO**  **(n=20)** | **Median (95% CI)**  **CP+BBO**  **(n=69)** | **Median (95% CI)**  **PDAC with Biliary Obstruction**  **(n=83)** | **Median (95% CI)**  **BBO plus CP with Biliary Obstruction**  **(n=27)** | **Median (95% CI)**  **PDAC without Biliary Obstruction**  **(n=44)** |
| CA19-9 | 130  (65.9–152.0) | 4  (3.0–5.0) | 16  (11.0–27.8) | 48.5  (13.2–184.2) | 19  (12.0–30.1) | 144  (83.3–169.4) | 42  (14.0–97.8) | 62  (33.1–141.8) |
| Eotaxin | 164  (145.5-183.9) | 98  (76.5–120) | 145  (116.1 – 190.2) | 137  (80.7–156.5) | 142.5  (117.5–161.6) | 164  (144.4–183.3) | 135.4  (90.5–146.3) | 165.1  (138.9–225.8) |
| IL-1b | 2.6  (2.4–3.0) | 1.6  (1.4–1.9) | 2  (1.8 – 2.2) | 1.3  (1.1–1.7) | 1.8  (1.6–2.0) | 2.74  (2.4–3.0) | 1.63  (1.3-1.8) | 2.45  (2.2–3.2) |
| IL-1ra | 146  (132.3–1.95.3) | 84  (61.8–106.6) | 143  (108–185.0) | 132  (82.8–222.2) | 133.7  (115.5–159.9) | 151.7  (132.9–200.5) | 132.16  (93.1–184.9) | 133.63  (105.0–229.6) |
| IL-6 | 12.3  (10.9–14.9) | 3.8  (3.3 – 5.2) | 9.9  (7.3 – 13.1) | 10.6  (7.7–15.7) | 9.9  (8.3–12.6) | 14  (11.5–17.4) | 11.36  (8.7–15.8) | 10.25  (8.3–12.5) |
| IL-8 | 24.7  (22.2–28.7) | 9.5  (8.1 – 10.0) | 16  (14.2 – 19.0) | 17  (12.2–32.2) | 16.6  (14.7–19.1) | 26  (21.8–32.2) | 18.6  (14.2–23.2) | 23.93  (19.2–27.9) |
| IP-10 | 1468  (1197–1785) | 444  (399.5 – 516.9) | 641  (563.1 – 811.1) | 533  (474.5–805.4) | 609  (544.0-718.2) | 1482  (1022.4–2288) | 579.2  (479.9–850.1) | 1451.34  (1134.3–1758.6) |
| MCP-1 | 40  (33.1–44.7) | 33  (24.9 – 40.9) | 45  (35.2 – 57.6) | 55  (35.5–97.6) | 50  (38.8–58.4) | 34.9  (29–43.8) | 53.3  (37.7–76.9) | 42.74  (35.7–53.3) |
| MIP-1b | 125  (108.9–140.0) | 85.7  (70.7 – 97.1) | 131  (100.6 – 165.7) | 102  (78.0–130.1) | 115  (99.4–141.9) | 132.5  (105–149.4) | 110.4  (86–165.4) | 144.295  (106.1–138.8) |
| PDGF | 17169  (15775–19531) | 14944  (11026–16638) | 14633  (11460–17821) | 7918  (3150–13431) | 13412  (9128-15444) | 17103  (15523–18429) | 9136  (5194–14849) | 18614.26  (14124-22410) |

Median circulating concentration (pg/mL) of cytokines with 95% confidence intervals. PDAC=Cancer, CP=Chronic Pancreatitis, BBO=Benign Biliary Obstruction. Wilcoxon signed rank tests were used to generate p-values for group comparisons. NS - non-significant.
