# Supplementary material for: Serum cytokine biomarker panels for discriminating pancreatic cancer from benign pancreatic disease
Source: Mol Cancer. 2014 May 20;13:114. doi: 10.1186/1476-4598-13-114 (PMC4032456; doi:10.1186/1476-4598-13-114)
Supplement: Additional file 2: Table S2 — Patient characteristics of Resectable and Advanced PDAC patients. [file 1476-4598-13-114-S2.docx]

**Additional file 2: Table S2.** Patient characteristics of Resectable and Advanced PDAC patients.

| **Training Set**  **n=84** | **Age (years) Median (95% CI)** | **Gender Male:Female** | **CA19-9 (KU/L) Median (95% CI)** | **Bilirubin (µmol/L) Median (95% CI)** | **Alive: Deceased** | **Survival (days) Median (95% CI)** | **Tumour Size (mm) Median (95% CI)** | **Moderately Differentiated** | **Poorly Differentiated** | **Well Differentiated** |
| --- | --- | --- | --- | --- | --- | --- | --- | --- | --- | --- |
| Resectable PDAC^t^ n=58 | 66  (64-69) | 31:27 | 131.5  (62.1-154.9) | 42.5  (26-72.5) | 7:51 | 409.5  (309-528) | 30  (29.2-35) | 31 | 16 | 10 |
| Advanced PDAC n=26 | 67  (63-71.5) | 16:10 | 153  (22.8-213) | 26  (9.5-39.8) | 1:25 | 139  (96.8-202) | NA | NA | NA | NA |
| **Test Set n=43** |  |  |  |  |  |  |  |  |  |  |
| Resectable PDAC^bc^ n=31 | 68  (68-71.8) | 18:13 | 120  (60.9-166.5) | 64  (23.5-120.7) | 3:27 | 420  (292-611) | 30  (24.9-35) | 14 | 10 | 5 |
| Advanced PDAC  N=12 | 68  (61-75) | 6:6 | 79.5  (30-189) | 18.5  (6-140) | 0:12 | 472.5 | NA | NA | NA | NA |

^t^data missing for 1 patient for tumour size and differentiation, ^b^data missing for 3 patients for tumour size, ^c^data missing for 1 patient for differentiation.
